# Prevalence of physical activity and obesity in US counties, 2001–2011: a road map for action

**DOI:** 10.1186/1478-7954-11-7

**Published:** 2013-07-10

**Authors:** Laura Dwyer-Lindgren, Greg Freedman, Rebecca E Engell, Thomas D Fleming, Stephen S Lim, Christopher JL Murray, Ali H Mokdad

**Affiliations:** 1 Institute for Health Metrics and Evaluation, University of Washington, 2301 5th Avenue, Suite 600, Seattle, WA, USA

**Keywords:** Physical activity, Obesity, Small area measurement, US counties

## Abstract

**Background:**

Obesity and physical inactivity are associated with several chronic conditions, increased medical care costs, and premature death.

**Methods:**

We used the Behavioral Risk Factor Surveillance System (BRFSS), a state-based random-digit telephone survey that covers the majority of United States counties, and the National Health and Nutrition Examination Survey (NHANES), a nationally representative sample of the US civilian noninstitutionalized population. About 3.7 million adults aged 20 years or older participated in the BRFSS from 2000 to 2011, and 30,000 adults aged 20 or older participated in NHANES from 1999 to 2010. We calculated body mass index (BMI) from self-reported weight and height in the BRFSS and adjusted for self-reporting bias using NHANES. We calculated self-reported physical activity—both any physical activity and physical activity meeting recommended levels—from self-reported data in the BRFSS. We used validated small area estimation methods to generate estimates of obesity and physical activity prevalence for each county annually for 2001 to 2011.

**Results:**

Our results showed an increase in the prevalence of sufficient physical activity from 2001 to 2009. Levels were generally higher in men than in women, but increases were greater in women than men. Counties in Kentucky, Florida, Georgia, and California reported the largest gains. This increase in level of activity was matched by an increase in obesity in almost all counties during the same time period. There was a low correlation between level of physical activity and obesity in US counties. From 2001 to 2009, controlling for changes in poverty, unemployment, number of doctors per 100,000 population, percent rural, and baseline levels of obesity, for every 1 percentage point increase in physical activity prevalence, obesity prevalence was 0.11 percentage points lower.

**Conclusions:**

Our study showed that increased physical activity alone has a small impact on obesity prevalence at the county level in the US. Indeed, the rise in physical activity levels will have a positive independent impact on the health of Americans as it will reduce the burden of cardiovascular diseases and diabetes. Other changes such as reduction in caloric intake are likely needed to curb the obesity epidemic and its burden.

## Background

Obesity and lack of physical activity are associated with several chronic conditions such as heart disease and diabetes, increased medical care costs, and premature death [1-3]. Obesity has increased rapidly during the past years; however, recent studies reported a decline in the rate of increase [4,5]. Recent studies reported a small increase in physical activity [6-8]. Levels of obesity and physical activity are likely to vary substantially across states and counties; different local governments have pursued a variety of approaches to address both risks. Understanding local trends in physical activity and obesity are important inputs to identifying successful and less successful strategies. Public health is local, and only local data will drive policy and action.

To examine the county-level changes in physical activity and obesity, we used data from the Behavioral Risk Factor Surveillance System (BRFSS) in the United States. We used data from the National Health and Nutrition Examination Survey (NHANES) to adjust BRFSS data for self-reporting bias in height and weight.

## Methods

The BRFSS is a state-based surveillance system that is operated by state health departments in collaboration with the Centers for Disease Control and Prevention. A detailed description of the survey methods is available elsewhere [9]. Briefly, the BRFSS collects data on many of the behaviors and conditions that place adults (aged ≥18 years) at risk for chronic disease. Trained interviewers collect data monthly, using an independent probability sample of households with telephones among the noninstitutionalized US adult population. All BRFSS methodology, questionnaires, and data are available at http://www.cdc.gov/brfss.

The NHANES is a nationally representative cross-sectional survey that collects data on self-reported health and also includes an examination component that collects an extensive array of biomarkers and anthropometric measures. We used data from the examination portion of the NHANES in the years 1999 to 2010, which produces national-level estimates every two years. NHANES data and questionnaires are available at http://www.cdc.gov/nchs/nhanes.htm. Additional information on the NHANES survey design is well documented elsewhere [9,10].

We used BRFSS data on self-reported weight and height to calculate body mass index (BMI) as weight (kg)/height^2^ (m^2^). Participants were classified as obese if their BMI ≥30 kg/m^2^. Self-reported weight and height were assessed by asking respondents, “About how much do you weigh without shoes?” and “About how tall are you without shoes?” To assess the prevalence of any leisure time physical activity, the BRFSS asked respondents, “During the past month, other than your regular job, did you participate in any physical activities or exercises such as running, calisthenics, golf, gardening, or walking for exercise?” In odd-numbered years the BRFSS included more detailed questions, which allows for distinguishing between insufficiently active and sufficiently active respondents. These questions recorded the amount of time and frequency of moderate and vigorous activity and were used to assess whether or not respondents met current physical activity guidelines. The exact methodology for collecting these data, as well as the physical activity guidelines used by BRFSS to define “sufficiently” active, changed in the 2011 survey compared to earlier years. Additional file 1 describes these changes in detail and presents the results of a sensitivity analysis that compares the estimated prevalence of sufficient physical activity under a variety of different definitions of recommended physical activity. For the 2011 survey we used the calculated variable provided by BRFSS to measure sufficient physical activity, while for the 2001 to 2009 surveys we recalculated this variable to match the definition used in 2011. Thus sufficient physical activity is defined as reporting 150 total minutes of moderate activity per week, the equivalent in vigorous activity, or combination of moderate and vigorous activity (1 minute vigorous activity = 2 minutes moderate activity). In 2011, BRFSS introduced a different methodology for its weights using raking [11] and included cellular telephones in its sample. Overall, data were available for 3,740,132 participants from all states and the District of Columbia. In 2010, the BRFSS covered 3,103 counties; in 2011, it covered 3,099.

The response rate in BRFSS has been declining through the years. This is a true for all types of surveys as concerns over privacy are increasing and people’s lifestyles are busier. For 2002 onwards the BRFSS provides all of its disposition codes and response rates according to the American Association for Public Opinion Research (AAPOR) definitions of response rates and reports using the Council of American Survey Research Organizations (CASRO) rates. CASRO is a measure of telephone survey operation, and it includes two components: 1) the proportion of numbers dialed where eligibility could be determined, and 2) the proportion of selected respondents who completed most or all of a survey once contacted. The BRFSS CASRO response rate is the number of interviews mostly or entirely completed as a proportion of all eligible households. To calculate the denominator for this response rate, it is assumed that the proportion of eligible telephone numbers among all telephone numbers where eligibility could not be determined is the same as among all telephone numbers where eligibility could be determined. This is a conservative estimate of the response rate as the proportion of these telephone numbers that are eligible is probably quite low because the BRFSS protocol requires 15 or more call attempts. The BRFSS cooperation rate is the proportion of all respondents identified as eligible who complete part or all of an interview. The CASRO varied from a cross-state median of 58.6% (range 42.2-82.6%) in 2002 to a median of 49.7% (range 33.8%-64.1%) in 2011. The cooperation rate varied from a median of 76.6% (range 62.5%-99.8%) in 2002 to a median of 73.8% (range 52.6%-84.4%) in 2011. All of the disposition codes, formulas, and response rates are available on the BRFSS website. The NHANES response rate for the examination portion of the interview has been more stable over time: 76% in 1999–2000, 80% in 2001–2002, 76% in 2003–2004, 77.4% in 2005–2006, 75.4% in 2007–2008, and 77.3% in 2009–2010.

The methods used to correct for self-report in calculating BMI and to estimate county-level prevalence of physical activity and obesity are similar to those presented in previously published work [12-14]. We correct for reporting biases in BMI by comparing BRFSS data, where height and weight are self-reported, to NHANES examination data, where height and weight are measured. For every two-year cycle of NHANES data and corresponding pooled two years of BRFSS data we calculated the mean BMI by sex and age (20–34, 35–44, 45–54, 55–64, 65–74, and 75+ years). We then regressed measured mean BMI from NHANES on reported mean BMI from BRFSS separately for males and females. We used the fitted coefficients from these models to calculate the corrected BMI for each individual represented in the BRFSS dataset and used this corrected BMI to assess whether or not each individual was obese.

We used previously described small area models to estimate the prevalence of obesity, any physical activity, and sufficient physical activity [14]. Specifically, we considered four logistic regression models for each outcome. The first model, the “Naïve” model, contains only individual-level covariates (race/ethnic group—white, black, Hispanic, Native American, and other—and age group), a linear time trend, and a county-level random effect. The second model, the “Covariate” model, includes everything in the Naïve model as well as a series of county-level covariates: for physical activity we included racial composition (National Center for Health Statistics bridged-race files), poverty (Census Bureau small area income and poverty estimates), pollution as measured by PM 2.5 (a measure of the concentration of fine particulate matter based on satellite imagery, ground-based sensors, and a chemical transport model) [15], percent rural (Census 2000 and 2010 SF1 files), percent of adults 25 years or older with a high school diploma (Census 2000 SF3 file and American Community Survey 2009–2011 five-year estimates), and percent of adults 16 years or older who are unemployed (Health Resources and Services Administration area resource file); for obesity we included all of the county-level covariates in the physical activity models as well as the number of doctors and dentists per 100,000 population (Health Resources and Services Administration area resource file). These covariates were included because we have reason to believe they may be related to obesity and/or physical activity and therefore useful for improving our predictions. Race, education, poverty, unemployment, and urban–rural status are well established social and economic covariates that are related to a wide variety of behaviors and health outcomes. We posit that pollution may be related to physical activity insofar as it may impact the environment in which individuals must choose whether or not to exercise. Finally, density of doctors and dentists was included in the obesity model as a proxy for contact with health systems which may influence people’s decisions regarding diet and other factors related to obesity. The third model, the “Geospatial” model, is the same as the Naïve model but contains a geospatial term that is calculated for each county as the mean of the posterior estimates of the county-level random effects from the Naïve model for all neighboring (i.e., adjacent) counties. Similarly, the fourth model, named the “Full” model, is the same as the Covariate model but contains a geospatial term calculated from the Covariate model. In the Covariate and Full model, both individual-level and county-level race variables are included. This is to account for both the potential direct effects of race (the effect of belonging to a particular race/ethnic group), as well as potential contextual effects (the effect of living in a county with a given racial composition). BRFSS data from 2001 to 2011 that were used to fit each model and observations where age, sex, race, or county were missing were excluded from all models, while observations where a given outcome was missing were excluded from the corresponding model. Finally, because we do not expect that changes in the prevalence of each outcome are necessarily linear over the entire study period, nor that the regional patterns will remain the same over the study period, we implemented a moving window approach wherein each model was fit on each set of five adjacent years of data (i.e., 2001–2005, 2002–2006, 2007–2011). Models were fit separately by sex and provided predictions for each county, year, sex, age group, and race group. We generated estimates for all races combined by calculating the population weighted average of the race-specific estimates using the race-specific populations in each country from the National Center for Health Statistics bridged-race files pooled over the study period. Finally, all estimates were age standardized using the 2000 census population as the age standard. We estimated uncertainty in all reported values using simulation methods.

For each outcome, we select among the four models using validation methods described in the works cited above. To summarize: counties where BRFSS records for at least 900 individuals are available from 2001 to 2005 were selected and a pooled gold standard was estimated using all data within these years. We then repeatedly sampled down these counties to 10, 50, and 100 individuals and fit all four models to the sampled-down data and compared the resulting estimates for these counties to the gold standard by calculating the concordance correlation, mean relative error, and root mean squared error. We found that the Full model performed best for all outcomes, and this model was used to derive all reported quantities. We also tested versions of the above four models that included marital status as an individual-level covariate; the performance of these models was generally similar or slightly worse, so we retained the more parsimonious models described above.

## Results

**Figure 1:**
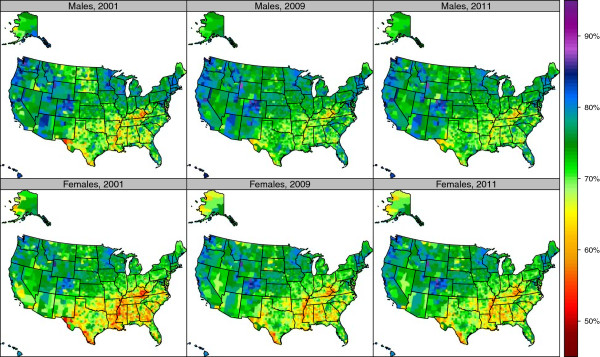
Age-standardized prevalence of reporting any physical activity by sex among adults age 20 and older, 2001, 2009, and 2011.

There was a wide variation in reporting any physical activity among US counties (Figure 1). Levels of physical activity were generally worse for men and women along the Texas-Mexico border, the Mississippi Valley, parts of the Deep South, and West Virginia. Table 1 identifies the counties with the highest and lowest rates of physical activity (Additional file 2 gives results for all counties). Douglas County, Colorado had the highest rate of any physical activity in the US (89.9%) for men in 2011, while Marin County, California had highest rate for women (89.5%). The county with the lowest rate for men was Wolfe County, Kentucky (54.7%), while for women it was McDowell County, West Virginia (50.9%). There was a wide variation between counties within a state in the level of any physical activity; for example in Virginia, the levels for men in 2011 varied from 85.1% in Arlington County to 57.7% in Dickenson County. For the US, there was no major change in the level of any physical activity from 2001 to 2009, although there was substantial variation across some counties.

**Table 1:** **Top and bottom 10 counties, by sex, for percent reporting any physical activity, percent reporting sufficient physical activity, and obesity prevalence (BMI ≥30 kg/m**^**2**^**), 2011**

**Top 10, Males**		**Bottom 10, males**		**Top 10, females**			**Bottom 10, females**
**Percent reporting any physical activity**							
Douglas, CO	89.9 (88.0, 91.7)	Wolfe, KY	54.7 (45.8, 62.9)	Marin, CA	89.5 (87.2, 91.3)	McDowell, WV	50.9 (45.6, 56.5)
Teton, WY	87.9 (84.6, 90.5)	McDowell, WV	54.9 (47.6, 61.8)	San Juan, WA	88.0 (85.8, 89.9)	Issaquena, MS	51.3 (44.0, 58.3)
Los Alamos, NM	87.7 (84.1, 90.6)	Owsley, KY	55.2 (46.1, 63.4)	Pitkin, CO	87.8 (84.9, 90.4)	Dunklin, MO	52.4 (46.0, 58.3)
Routt, CO	87.1 (83.7, 89.7)	Issaquena, MS	57.0 (48.1, 65.1)	Routt, CO	87.5 (84.5, 89.8)	Wolfe, KY	53.8 (46.3, 60.6)
Marin, CA	86.9 (83.7, 89.7)	Clinton, KY	57.6 (48.8, 65.8)	Teton, WY	86.9 (84.4, 89.1)	Owsley, KY	54.0 (46.6, 61.2)
Kauai, HI	86.8 (84.0, 89.1)	Dickenson, VA	57.7 (49.7, 65.6)	Douglas, CO	86.3 (84.5, 88.1)	East Carroll, LA	54.0 (47.2, 61.0)
Summit, UT	86.7 (84.1, 89.0)	Mingo, WV	57.9 (51.7, 64.3)	Santa Cruz, CA	85.7 (82.9, 88.2)	Pemiscot, MO	54.0 (47.7, 60.5)
San Juan, WA	86.6 (83.6, 89.2)	Holmes, OH	58.2 (49.7, 67.0)	Island, WA	85.7 (83.3, 87.7)	Lee, AR	54.1 (47.5, 60.8)
Orange, NC	86.5 (83.7, 88.8)	Leslie, KY	58.6 (49.7, 66.8)	Summit, UT	85.5 (83.1, 87.5)	Mississippi, MO	54.2 (46.8, 61.0)
Island, WA	86.4 (83.7, 89.0)	Starr, TX	58.8 (50.1, 66.6)	Summit, CO	85.5 (81.6, 88.3)	La Salle, TX	54.3 (47.0, 61.1)
**Percent reporting sufficient physical activity**							
Teton, WY	77.5 (72.0, 82.4)	Owsley, KY	33.1 (24.8, 42.6)	Routt, CO	74.7 (70.2, 78.7)	Issaquena, MS	28.4 (22.5, 35.0)
Summit, UT	73.2 (68.0, 77.3)	Holmes, OH	33.7 (25.4, 42.6)	Marin, CA	74.2 (69.8, 78.3)	Noxubee, MS	29.0 (22.6, 35.9)
Routt, CO	72.9 (66.9, 78.4)	Wolfe, KY	34.2 (25.6, 44.3)	Teton, WY	72.7 (67.9, 76.7)	Quitman, MS	29.1 (22.7, 35.5)
Summit, CO	72.7 (65.2, 79.0)	Issaquena, MS	34.6 (26.1, 44.2)	Pitkin, CO	72.4 (66.8, 77.7)	Tallahatchie, MS	30.7 (24.8, 37.7)
Jefferson, WA	72.2 (66.0, 77.8)	McDowell, WV	34.7 (27.0, 43.2)	San Juan, WA	71.6 (67.5, 75.5)	Haywood, TN	30.7 (24.3, 37.5)
Nevada, CA	71.9 (64.9, 78.0)	Casey, KY	34.8 (27.7, 43.2)	Summit, UT	69.6 (65.6, 73.5)	Tunica, MS	30.7 (24.2, 37.6)
La Plata, CO	71.9 (66.2, 76.9)	Clay, KY	35.8 (27.9, 45.3)	Eagle, CO	69.6 (64.6, 75.0)	McDowell, WV	30.8 (25.4, 37.1)
Wasatch, UT	71.7 (67.0, 76.1)	Mingo, WV	36.0 (29.3, 43.9)	Barnstable, MA	69.2 (65.4, 72.7)	Humphreys, MS	30.9 (24.7, 38.4)
Kauai, HI	71.6 (66.9, 75.8)	Clinton, KY	36.1 (27.2, 45.8)	Benton, OR	69.1 (63.8, 74.3)	East Carroll, LA	31.2 (25.2, 38.7)
Los Alamos, NM	71.4 (64.2, 77.3)	Taliaferro, GA	36.4 (27.7, 46.3)	Rio Blanco, CO	68.8 (61.3, 75.1)	Taliaferro, GA	31.3 (25.0, 38.2)
**Percent obese (BMI ≥30 kg/m**^2^**)**							
San Francisco, CA	18.3 (16.4, 22.2)	Owsley, KY	46.9 (41.0, 53.4)	Falls Church City, VA	17.6 (13.8, 21.3)	Issaquena, MS	59.3 (52.5, 64.9)
New York, NY	19.1 (16.8, 22.2)	Issaquena, MS	46.7 (40.4, 53.4)	Pitkin, CO	18.5 (15.1, 21.9)	Humphreys, MS	59.1 (52.7, 64.4)
Falls Church City, VA	19.5 (15.6, 23.7)	East Carroll, LA	46.6 (40.5, 52.8)	Douglas, CO	18.6 (16.5, 20.9)	East Carroll, LA	58.9 (52.1, 64.2)
Santa Fe, NM	21.0 (18.9, 24.1)	Holmes, OH	46.4 (40.2, 52.8)	Routt, CO	19.0 (15.9, 22.0)	Quitman, MS	58.1 (51.8, 63.8)
Pitkin, CO	21.3 (17.9, 26.0)	Starr, TX	46.2 (39.6, 52.5)	Teton, WY	19.6 (16.7, 22.5)	Greene, AL	58.0 (51.0, 63.7)
Teton, WY	21.6 (18.6, 25.1)	Lewis, KY	46.1 (41.7, 51.7)	Summit, UT	20.0 (17.4, 22.7)	Allendale, SC	58.0 (51.6, 63.9)
Eagle, CO	22.0 (18.9, 26.5)	McDowell, WV	46.0 (40.4, 51.5)	San Francisco, CA	20.9 (17.8, 23.7)	Wilcox, AL	57.8 (51.0, 63.5)
Fairfax City, VA	22.0 (17.7, 26.4)	Lincoln, WV	45.9 (40.3, 51.8)	Eagle, CO	20.9 (17.3, 24.0)	Shannon, SD	57.7 (50.2, 64.0)
District of Columbia	22.4 (20.6, 24.8)	Allen, LA	45.6 (39.8, 50.9)	Marin, CA	21.1 (17.5, 23.7)	Jefferson, MS	57.7 (51.0, 63.7)
Summit, UT	22.4 (20.0, 26.5)	Union, FL	45.5 (41.3, 50.3)	Gallatin County Yellowstone Park, MT	21.9 (19.5, 24.4)	Holmes, MS	57.6 (52.2, 62.0)

**Figure 2:**
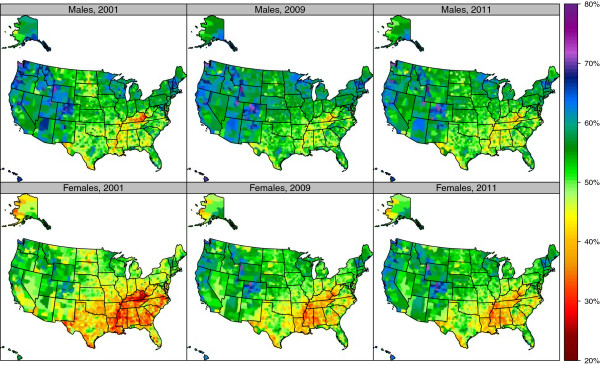
Age-standardized prevalence of reporting sufficient physical activity by sex among adults age 20 and older, 2001, 2009, and 2011.

**Figure 3:**
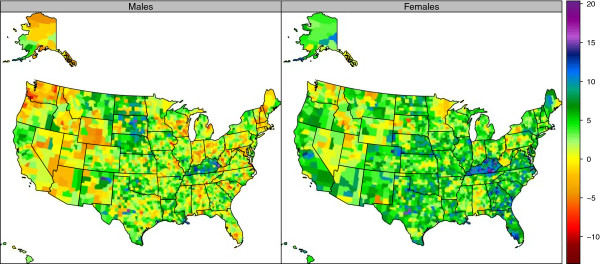
Percentage change in age-standardized prevalence of reporting sufficient physical activity by sex among adults age 20 and older, 2001–2009.

Reporting of sufficient physical activity also varied widely (Figure 2, Table 1, and Additional file 3). For men in 2011, Teton County, Wyoming had the highest reported prevalence of sufficient physical activity (77.5%), and Owsley County, Kentucky had the lowest (33.1%); Routt County, Colorado had the highest (74.7%), and Issaquena County, Mississippi had the lowest (28.4%) for women in 2011. There was a wide variation between counties within a state. For example, in Colorado, prevalence of sufficient physical activity among women varied from a high of 74.7% in Routt County to 42.7% in Crowley County in 2011. In contrast to reporting of any physical activity, our results showed an increase in the prevalence of reporting sufficient physical activity from 2001 to 2009 (Figure 3 and Table 2) in a number of communities. While levels of sufficient physical activity are generally higher in men than in women, increases between 2001 and 2009 were greater in women than men. Counties in Kentucky, Nebraska, Montana, Florida, Georgia, and parts of California reported the largest gains. The greatest increase in sufficient physical activity for men was observed in Concho County, Texas, with an increase from 41.4% in 2001 to 58.2% in 2009, a 16.7 (5.7-27.2) percentage point increase. The greatest increase in sufficient physical activity for women was seen in Morgan County, Kentucky, with an increase from 25.7% in 2001 to 44.0% in 2009, an 18.3 (11.6-25.3) percentage point increase.

**Table 2:** **Top and bottom 10 counties, by sex, for change in percent reporting any physical activity, percent reporting sufficient physical activity, and obesity prevalence (BMI ≥30 kg/m**^**2**^**), 2001-2009**

**Top 10, Males**		**Bottom 10, males**		**Top 10, females**		**Bottom 10, females**	
**Change in percent reporting any physical activity**							
Concho, TX	16.2 (7.4, 25.1)	Juneau City, AK	−7.5 (−10.3, -4.2)	Concho, TX	13.3 (4.2, 21.9)	Dewey, SD	−9.6 (−18.0, -1.2)
Martin, KY	14.6 (4.9, 24.9)	Fond du Lac, WI	−7.1 (−12.8, -1.5)	Emporia City, VA	12.5 (3.7, 21.2)	Shannon, SD	−7.4 (−16.6, 1.4)
Floyd, KY	12.5 (5.1, 19.4)	Cabell, WV	−7.1 (−12.2, -2.1)	Candler, GA	11.5 (3.3, 19.8)	Cabell, WV	−7.3 (−12.1, -2.6)
Harrisonburg City, VA	11.3 (4.1, 18.8)	Dickenson, VA	−6.9 (−16.3, 2.5)	Banks, GA	11.4 (3.0, 19.9)	Lincoln, WV	−6.7 (−14.1, 1.0)
St. Martin, LA	10.9 (2.8, 18.2)	Carbon, WY	−6.7 (−11.9, -1.3)	Evangeline, LA	11.0 (3.6, 18.5)	Gallia, OH	−6.4 (−14.2, 1.3)
Sheridan, ND	10.7 (1.6, 20.1)	York, NE	−6.7 (−12.0, -1.0)	West Feliciana, LA	10.7 (1.9, 19.6)	Jackson, OH	−6.4 (−14.0, 1.8)
Schleicher, TX	10.6 (2.1, 19.4)	Meade, SD	−6.5 (−11.2, -1.8)	Schleicher, TX	10.7 (2.3, 19.2)	Bristol Bay, AK	−6.2 (−13.2, 0.0)
Candler, GA	10.6 (1.2, 19.3)	Dodge, WI	−6.5 (−12.4, -0.5)	Union, TN	10.6 (1.0, 19.8)	Grant, IN	−6.1 (−12.2, 0.3)
Childress, TX	10.4 (2.8, 17.9)	Lander, NV	−6.4 (−14.9, 1.3)	Hancock, TN	10.3 (0.6, 20.1)	Delaware, IN	−6.0 (−12.0, -0.4)
East Carroll, LA	10.3 (0.1, 19.8)	Chemung, NY	−6.4 (−13.0, -0.2)	Childress, TX	10.1 (1.5, 18.1)	Hill, MT	−5.9 (−9.9, -2.0)
**Change in percent reporting sufficient physical activity**							
Concho, TX	16.7 (5.7, 27.2)	Virginia Beach City, VA	−11.4 (−19.2, -4.0)	Morgan, KY	18.3 (11.6, 25.3)	Cabell, WV	−6.2 (−12.8, 0.3)
Pike, KY	15.9 (9.0, 22.9)	Cowlitz, WA	−10.0 (−16.9, -2.3)	McCreary, KY	18.2 (10.7, 25.6)	Dewey, SD	−6.0 (−15.5, 3.8)
Elliott, KY	15.9 (5.8, 26.1)	Petersburg City, VA	−9.3 (−20.0, 1.8)	Manassas Park City, VA	18.0 (8.5, 28.1)	Camas, ID	−5.7 (−16.1, 5.0)
Faulk, SD	15.0 (4.2, 26.0)	Marion, WV	−8.5 (−16.4, -0.5)	Owen, KY	17.6 (7.6, 26.4)	Monongalia, WV	−5.6 (−13.2, 1.5)
McCreary, KY	14.9 (5.1, 23.8)	Fairfax City, VA	−8.5 (−16.9, 1.6)	Pulaski, KY	17.2 (10.8, 23.3)	Miami, IN	−5.4 (−14.5, 3.8)
Martin, KY	14.8 (5.5, 23.6)	Johnson, IA	−8.4 (−15.2, -1.1)	Perquimans, NC	16.9 (8.1, 25.6)	Mercer, PA	−5.4 (−13.9, 2.3)
Mora, NM	14.3 (4.1, 25.0)	Richland, SC	−8.0 (−13.8, -2.2)	Edmonson, KY	16.7 (7.6, 25.9)	Lawrence, SD	−5.2 (−11.6, 1.3)
Muhlenberg, KY	13.7 (4.3, 22.3)	Bristol, RI	−7.6 (−14.2, 0.1)	Concho, TX	16.5 (7.0, 26.2)	Harrisonburg City, VA	−5.0 (−15.3, 4.7)
Bond, IL	13.3 (2.9, 24.0)	Norfolk City, VA	−7.6 (−15.5, 0.5)	Elliott, KY	16.1 (7.0, 24.9)	Porter, IN	−4.9 (−12.0, 2.8)
Ohio, KY	12.7 (2.8, 22.4)	Columbia, OR	−7.5 (−15.3, 1.0)	Knox, KY	15.5 (8.3, 22.2)	Otero, NM	−4.8 (−11.4, 1.1)
**Change in percent obese (BMI ≥30 kg/m**^**2**^**)**							
Buffalo, SD	−2.9 (−11.4, 5.3)	Lewis, KY	15.8 (9.5, 22.0)	Keweenaw, MI	−1.4 (−6.8, 7.1)	Berkeley, SC	16.4 (11.8, 20.2)
Ziebach, SD	−2.8 (−10.9, 5.8)	Webb, TX	14.6 (8.5, 20.5)	Rio Blanco, CO	−1.4 (−6.7, 4.7)	Crowley, CO	14.2 (6.6, 22.2)
Roosevelt, MT	−0.9 (−7.3, 6.2)	Allen, LA	14.2 (6.7, 20.0)	Routt, CO	−0.5 (−4.6, 3.9)	Ionia, MI	14.1 (6.9, 19.9)
Corson, SD	−0.6 (−7.7, 7.4)	Allen, OH	14.1 (7.6, 20.3)	Pitkin, CO	−0.2 (−4.6, 4.4)	Barry, MI	13.9 (7.9, 19.9)
Daniels, MT	0.0 (−6.7, 7.1)	Tazewell, VA	14.1 (7.5, 20.6)	Red Lake, MN	0.1 (−6.8, 7.8)	Hancock, WV	13.8 (7.7, 19.6)
Staunton City, VA	0.2 (−5.3, 8.8)	Zapata, TX	14.0 (5.8, 21.7)	Eagle, CO	0.2 (−4.2, 4.5)	Owsley, KY	13.6 (5.6, 22.0)
Menominee, WI	0.2 (−7.8, 8.7)	Salem, NJ	13.8 (8.1, 19.3)	La Plata, CO	0.4 (−3.8, 4.9)	Lee, SC	13.5 (6.8, 19.7)
McCreary, KY	0.3 (−6.4, 7.8)	Ottawa, OH	13.4 (5.5, 19.3)	Archuleta, CO	0.5 (−4.5, 6.2)	Allen, OH	13.3 (7.3, 19.4)
Glacier, MT	0.5 (−6.1, 7.7)	Dallas, IA	13.2 (8.0, 19.3)	Chaffee, CO	0.6 (−4.4, 5.7)	Calhoun, FL	13.1 (7.6, 17.8)
Apache, AZ	0.5 (−5.8, 7.3)	Cambria, PA	13.2 (6.3, 18.8)	Marion, AL	0.7 (−5.3, 7.1)	Crittenden, AR	13.1 (8.4, 19.5)

**Figure 4:**
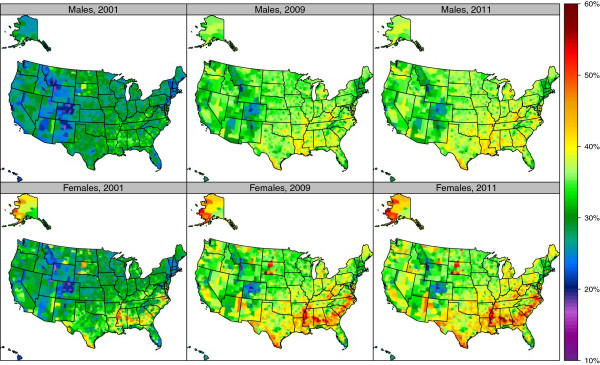
**Age-standardized prevalence of obesity (BMI ≥30 kg/m**^**2**^**) by sex among adults age 20 and older, 2001, 2009, and 2011.**

**Figure 5:**
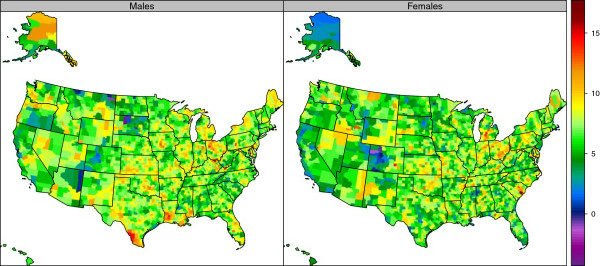
**Percentage change in age-standardized prevalence of obesity (BMI ≥30 kg/m**^**2**^**) by sex among adults age 20 and older, 2001–2009.**

The prevalence of obesity varied widely by counties (Figure 4, Table 1, and Additional file 4). In 2011, the highest rates for men were observed in Owsley County, Kentucky (46.9%) and for women, Issaquena County, Mississippi (59.3%). The lowest obesity rates for men were observed in San Francisco County, California (18.3%) and for women in Falls Church City, Virginia (17.6%). There was a wide variation in obesity levels by counties within a state. For example, in Virginia the prevalence of obesity for women was 17.6% in Falls Church City compared to 55.4% in Petersburg City. Obesity prevalence increased in US counties from 2001 to 2009 (Figure 5 and Table 2). The greatest increase for men was observed in Lewis County, Kentucky, with a change from 28.9% in 2001 to 44.7% in 2009, a 15.8 (9.5-22.0) percentage point increase. For women, the greatest increase was observed in Berkeley County, South Carolina, with a change from 31.6% in 2001 to 47.9% in 2009, a 16.4 (11.8-20.2) percentage point increase. The greatest decrease in obesity prevalence for women was observed in Keweenaw County, Michigan with a −1.4 (−6.8-7.1) percentage point change. For men, the greatest decrease in obesity prevalence was observed in Buffalo County, South Dakota, with a −2.9 (−11.4-5.3) percentage point change. Obesity prevalence decreased in only nine counties—five for men and four for women—and in none of these counties was the change statistically significant.

**Figure 6:**
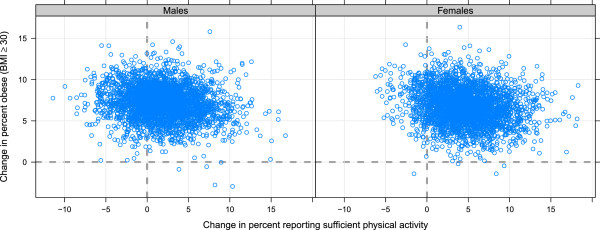
Relationship between change in prevalence of obesity and change in prevalence of sufficient physical activity by sex in adults age 20 and older, 2001–2009.

There was a low correlation between change in the level of physical activity and obesity in US counties (Figure 6). From 2001 to 2009, for every 1 percentage point increase in physical activity, obesity prevalence was 0.11 percentage points lower. This result is robust when controlling for a number of other covariates. Table 3 shows the results of a regression of change in obesity on change in physical activity controlling for percent rural, change in poverty, change in unemployment, change in number of doctors per 100,000 population, and baseline level of obesity in 2001.

**Table 3:** Regression parameters from regression relating change in obesity to change in physical activity

**Parameter**	**Estimate**	**SE **
Intercept	7.386	0.1902*
Change in physical activity, 2001-2009	−0.107	0.0072*
Percent rural in 2009	0.002	0.0009
Change in poverty, 2001-2009	0.059	0.0103*
Change in unemployment, 2001-2009	0.089	0.0140*
Change in number of doctor’s per 100,000 population, 2001-2009	−0.005	0.0007*
Obesity prevalence in 2001	−0.010	0.0067

* Significant at the 0.001 level.

We have included a web appendix for all levels of obesity, any physical activity, and sufficient physical activity at the county level for all years of our study (Additional files 2, 3, and 4).

## Conclusions

Our study revealed a wide variation in obesity and physical activity levels among counties in the US. To our knowledge, this is the first study to combine data from NHANES and BRFSS in order to adjust for self-reporting bias of weight and height to measure obesity at the county level. The data showed that although levels of physical activity likely increased during the 2000s, the level of obesity kept increasing in nearly all counties. The US Burden of Diseases, Injuries, and Risk Factors Study [17] suggests that in 2010 physical inactivity and low physical activity accounted for 234,000 deaths and 5.2% of disability-adjusted life years independent of BMI. Our results call for focusing on a message of the health benefits of physical activity instead of a means for weight reduction.

Elevated BMI is associated with multiple outcomes including higher rates of ischemic heart disease, stroke, cardiomyopathies, hypertensive heart disease, atrial fibrillation, diabetes, osteoarthritis, low back pain, chronic kidney disease, colorectal cancer, breast cancer, esophageal cancer, kidney cancer, gallbladder cancer, pancreatic cancer, and uterine cancer [17]. A substantial component of the cardiovascular effects of elevated BMI operate through blood cholesterol and blood pressure [13]. Because of the increase in obesity over the last two decades, the US Burden of Disease shows that high BMI is now the third-leading risk factor in terms of attributable disability-adjusted life years [17]. The public attention and awareness of the adverse consequences of obesity may have led the population to modify the composition of their diets and increase physical activity. However, these efforts at this time have not made an impact on the epidemic of obesity. Indeed, to address the epidemic of obesity in the US a comprehensive approach may be needed. Although the evidence on successful programs is very limited, reducing caloric intake will likely require community changes as well as individual behavioral response.

During the past decade, there has been no overall improvement in the percentage of adults reporting any physical activity in the BRFSS: for men, the rate was 22.5% in 2001 and 22.4% in 2011; for women, the rate was 28.1% in 2001 and 25.9% in 2011. New strategies to target this segment of the population must be proposed and tested if continued progress in increasing physical activity is to be sustained across the country.

The success of improving levels of sufficient physical activity by a large margin in selected communities stands out in marked contrast to the failure to observe any statistically significant reductions in obesity in any county. Of the 10 counties with the largest improvements, six for men and seven for women were in Kentucky. Other areas with substantial improvements in sufficient physical activity include the metropolitan areas around Atlanta, Los Angeles, San Francisco, San Diego, Houston, and Denver. Although overall there is no correlation between improvements and county size, some large urban areas have been successful. This analysis does not identify why efforts to promote physical activity in these communities have been so much more successful than elsewhere in the country. Further in-depth analyses of these local experiences may be helpful in identifying program insights that can be transferred to other communities, although in-migration to some urban areas may also be a factor. The success in increasing physical activity in some urban and rural communities suggests that more progress in increasing physical activity can be made across the country. This progress, however, will not on its own reverse increases in obesity.

Our findings on increasing obesity levels and improved levels of physical activity are puzzling when put in context with reported declines of mean adult caloric intake in NHANES, from 2,269 kcal/day in 2003–2004 to 2,195 kcal/day in 2009-2010 [18]. These self-reported figures for caloric consumption are markedly lower than average caloric availability in the US, which exceeds 3,750 kcal/day [19]. The increases in obesity, decreases in caloric intake, and increases in physical activity seen here require some explanation. First, reported caloric intake in NHANES is known to be biased downwards [12]. When the data from 24-hour diet recall were validated, obese individuals were more likely to underestimate their caloric intake [20,21]. It is possible that as obesity has increased, caloric reporting may have been further underestimated. Alternatively, reporting bias may have increased over time due to social attention on obesity and total caloric intake. Second, if caloric intake was substantially more than the level required for energy balance, the reported 74 kcal/day reduction in intake would indicate the population was not in energy balance, despite higher levels of physical activity. Third, the increase in self-reported physical activity could also be due to increased positivity bias. Given increased public awareness campaigns for physical activity, it is possible that individuals have become more likely to report positive behaviors even if they have not increased their physical activity. Our sensitivity analyses of different ways of constructing sufficient physical activity show that the national trend may be leveling rather than increasing. Finally, it is possible that the behaviors of residents in urban settings are different from those in rural areas. Thus, since NHANES is mainly conducted in about 15 large urban areas in 2011, it is possible that NHANES data are more reflective of urban areas rather than rural areas.

Our findings have some limitations. First, NHANES does not release county identifiers, and we were not able to use such a variable in our adjustment. We assumed that the same correlation between self-reported weight and true weight from a national sample applies to all counties. Our correction model assumes that misreporting of height and weight do not vary over time or by location. However, even if the changes varied by time, our results on the variation in obesity prevalence across counties would not be affected. Second, BRFSS introduced a change in its methodology for weighting in 2011 and included cellular telephones for the first time. Additionally, BRFSS revised the questions used to assess a respondent’s physical activity levels and also changed the standard for recommended physical activity to which respondents were compared. To deal with these changes to how sufficient physical activity was measured and defined we have recalculated this variable for all years to apply the definition used in the 2011 BRFSS. Further, we consider trends over from 2001 to 2009 rather than 2001 to 2011 so that the reported trends will not be influenced by the changes in survey methodology in the 2011 survey. We reported the 2011 prevalence to provide a baseline for the future using the new definition and to account for BRFFS methodology change. Our sensitivity analysis (Additional file 1), however, shows that our finding that some communities have achieved major increases in prevalence of sufficient physical activity is robust to the definition of sufficient physical activity employed. Third, BRFSS response rates decreased over our study period. However, BRFSS has always been reliable and valid when compared to other household surveys [9,16]. Fourth, our physical activity estimates are based on self-reports; direct measures of energy expenditure at the national level are not available to validate self-report. Finally, this study is an area-level analysis; we are not testing hypotheses about the determinants of individual behavior or outcomes. Rather, we are examining the relationships between community characteristics and community outcomes. Further, while we report the association between changes in physical activity and obesity prevalence, controlling for a number of key variables, there may still be other variables that confound the relationship between change in physical activity and change in obesity. Residual measurement error in physical activity levels could also attenuate the estimated relationship between change in physical activity and change in obesity.

Despite these limitations, our study has several advantages. Our study is based on a large sample size. Moreover, our small area estimation method has an advantage compared to other approaches, because it allows us to validate its performance in simulation studies using counties with large sample sizes. Despite these improved small area methods, estimates for some counties with small numbers of responses have wide uncertainty intervals, thereby making detection of statistically significant change over time difficult. Finally, we adjusted for self-reported bias in obesity levels.

Our findings call for searching for more aggressive strategies to prevent and control obesity. Similar to tobacco prevention and control, multisectorial coordinated actions involving our health care and public health systems, along with other government departments such as agriculture, education, and transportation, and non-governmental organizations including consumer groups, service associations, professional bodies, and laws may be needed. Consideration should be given to the role of food labeling, taxation, and incentives both for individuals and for communities [22-24]. A balance between caloric intake (consumption) and physical activity levels (expenditure) is needed so that increasing physical activity is not negated by increasing caloric intake. The NHANES analysis of caloric trends gives hope that caloric intake may have stopped increasing; the challenge will be to reduce caloric intake and simultaneously continue increasing physical activity.

The geographic distribution of obesity and physical inactivity are of great importance to public health policy at the local level. Indeed, public health is local and our data will empower counties to design, implement, and evaluate public health programs to address these risk factors. Moreover, county-level information can empower the public to act. Ultimately, our data will allow us to learn from successful programs and improve the efficiency of others dealing with physical inactivity and obesity.

## Competing interests

The authors declare that they have no competing interests.

## Authors’ contributions

CJLM, LDL, GF, TF, and RE developed and applied the model to estimate physicial activity and obesity for sex and race by county. AHM and CJLM designed the overall study and analytical strategy. AHM and CJLM wrote the first draft. AHM, CJLM, and LDL revised the paper. All authors have read and approved the final manuscript.

## Supplementary Material

Additional file 1 Definition of recommended physical activity in the BRFSS and methodology with prevalence of sufficient physical activity with different definitions of physical activity and change in sufficient physical activity prevalence by state with different definitions of physical activity. Click here for file

Additional file 2 Prevalence of reported physical activity (any) for all counties, 2001–2011. Click here for file

Additional file 3 Prevalence of reported physical activity (meeting recommended guidelines) for all counties, 2001–2011. Click here for file

Additional file 4 **Prevalence of obesity (BMI ≥30 kg/m**^**2**^**) for all counties, 2001–2011.** Click here for file
