# Supplementary material for: Prevalence of physical activity and obesity in US counties, 2001–2011: a road map for action
Source: Popul Health Metr. 2013 Jul 10;11:7. doi: 10.1186/1478-7954-11-7 (PMC3718620; doi:10.1186/1478-7954-11-7)

Additional File 1:
Prevalence of Physical activity and obesity in US counties 2001-2011: A Road Map for Action.

Definition of Recommended Physical Activity in the BRFSS

In odd numbered years BRFSS collects more detailed data on physical activity which allows for calculation of the prevalence of achieving recommended levels of physical activity; the BRFSS datasets in these years include calculated variables which indicate the physical activity level for each respondent which were utilized in this analysis for the purpose of classifying respondents as sufficiently active or insufficiently active.

For the 2001 to 2009 surveys the calculated variable _RFPAREC categorizes respondents into three groups: those who meet recommendations ("Respondents that report doing enough moderate or vigorous physical activity to meeting the recommendations"), those who are insufficiently active ("Respondents that report doing insufficient moderate or vigorous physical activity to meet recommendations"), and those who are inactive ("Respondents that report doing no moderate or vigorous physical activity"). For the purposes of this analysis, we partitioned respondents into two groups: those who meet recommendations (the first group) and those who do not (the second and third groups). This calculated variable was based on questions regarding moderate and vigorous physical activity. Respondents were first asked if they participated in any moderate physical activities outside of work ("…such as brisk walking, bicycling, vacuuming, gardening, or anything else that causes some increase in breathing or heart rate") for at least 10 minutes at a time in a usual week and, if so, how many days and for how long each day. To be classified as meeting recommended goals for moderate activity, a respondent needed to report 5 or more days of moderate activity with 30 or more minutes per day. Respondents were then asked if they participated in any vigorous physical activities outside of work ("…such as

running, aerobics, heavy yard work, or anything else that causes large increases in breathing or heart rate") for at least 10 minutes at a time in a usual week and, if so, how many days and for how long each day. To be classified as meeting recommended goals for vigorous activity, a respondent needed to report 3 or more days of vigorous activity with 20 or more minutes per day. An individual who met the moderate goal, the vigorous goal, or both was classified as meeting recommendations while an individual who reported either moderate activity, vigorous activity, or both but didn't meet the goals for either moderate or vigorous activity was classified as insufficiently active. In the 2001 to 2009 surveys these detailed questions on physical activity were asked in a separate section than the question on any physical activity—"During the past month, other than your regular job, did you participate in any physical activities or exercises such as running, calisthenics, golf, gardening, or walking for exercise"—used to classify respondents as active or inactive for the purposes of measuring prevalence of any physical activity. This question was not used as a filter for the more detailed questions: respondents were asked about moderate and vigorous activity whether or not they reported any physical activity in this earlier question.

For the 2011 survey the calculated variable _PACAT which categorizes respondents into four groups: highly active ("Respondents who reported doing enough physical activity to meet the 300-minute (or vigorous equivalent) aerobic recommendation"), active ("Respondents who reported doing 150-300 minutes (or vigorous equivalent) of physical activity"), insufficiently active ("Respondents who reported doing insufficient physical active (11-149 minutes)"), and inactive ("Respondents who reported doing no physical activity"). For the purposes of the present analysis, we divided respondents into two groups: those who meet recommendations (the

first two groups) and those who did not (the second two groups). For the 2011 survey the questions on which the calculated variable is based changed considerably compared to the older surveys. The question used to classify respondents as active or inactive ("During the past month, other than your regular job, did you participate in any physical activities or exercise such as running, calisthenics golf, gardening, or walking for exercise") was moved to the same section as the more detailed physical activity questions and, unlike in previous surveys, was used as a filter: individuals who answered no to this question were not asked the more detailed questions about physical activity. Respondents who answered yes to this first question were subsequently asked about the type of physical activity they spent the most time doing, how many times in a week (or month) they took part in this physical activity, and for how long each time. These questions were repeated for the activity that the person spent the next most amount of time on. Each activity was subsequently classified as moderate or vigorous based on an assigned MET value and a respondent's assumed functional capacity based on their age and sex and then the total number of moderate or vigorous minutes of activity per week was calculated. To calculate moderate or vigorous equivalent minutes of activity per week, minutes performing a vigorous activity were multiplied by 2 and added to minutes performing a moderate activity. Respondents were classified as highly active if the moderate or vigorous equivalent minutes of activity exceeded 300, active if above 150 but below 300, insufficiently active for more than 10 but less than 150 minutes, and inactive if they reported less than 10 minutes of activity in this section, or responded no to the question about any physical activity.

The 2011 survey differs from the 2001-2009 survey in several ways that could affect comparability of the estimates derived from these surveys. First, what constitutes meeting recommended goals for physical activity differs in three important ways:

(1) The total amount of time for vigorous activity increased from 60 minutes in the 2001 to 2009 surveys to 75 minutes in the 2011 survey.

(2) In order to meet the recommended goals in the 2001 to 2009 surveys, respondents needed to report at least 3 days of vigorous activity or 5 days of moderate activity in addition to reporting a sufficient number of minutes overall; in contrast, in the 2011 survey only the total number of minutes was considered.

(3) In the 2001 to 2009 surveys a respondent had to meet either the moderate of the vigorous goal independently in order to meet the overall goal, whereas in the 2011 survey moderate and vigorous physical activity could be combined in order to meet the goal.

Second, the 2001 to 2009 surveys do not use the question on any physical activity as a filter, while the 2011 survey does. This means that in the 2001-2009 survey the responses to the any physical activity question can be, and often are, inconsistent with individuals who answer no to the any physical activity question later reporting sufficient moderate or vigorous activity to meet recommendations, while in the 2011 survey by design a respondent's classification as active or inactive is always consistent with their classification by level of activity (though individuals who report that they are active but report less than 10 minutes total activity are ultimately classified as inactive). Finally, the manner in which minutes of moderate and vigorous activity are estimated differs: in the 2001 to 2009 surveys, respondents make the decision about what qualifies as a moderate or vigorous activity and report on total time for each level of activity; in the 2011

survey respondents report on only the two activities they engage in most often and these are later classified as either moderate or vigorous in a systematized fashion.

We assess the impact of the differences in the 2001 to 2009 surveys compared to the 2011 survey by examining trends at the national level in prevalence of sufficient activity based on a series of different definitions of sufficiently active and compare these to the trend observed using the calculated variables described above (green line, appendix figure 1). Specifically, we calculate prevalence of sufficient activity for all surveys based on three definitions:

(1) 150 minutes of total moderate physical activity and/or 60 minutes of total vigorous physical activity (solid orange line, appendix figure 1). For the 2001 to 2009 surveys this allows us to assess the impact of considering total minutes rather than requiring a certain number of days and a certain duration each day.

(2) 150 minutes of total moderate physical activity and/or 75 minutes of total vigorous physical activity (solid purple line, appendix figure 1). For the 2001 to 2009 surveys this allows us to assess both the impact of considering total minutes, as with option (1), but also the impact of increasing the vigorous goal from 60 to 75 minutes.

(3) 150 minutes of total moderate/vigorous equivalent physical activity (solid pink line, appendix figure 1). This is the definition used in the 2011 survey. For the 2001 to 2009 survey this allow us to assess both the impact of considering total minutes and the increase in the threshold for vigorous activity, as with option 2, and also the impact of allowing for the moderate and vigorous activity to be combined to meet recommendations.

Additionally, to assess the impact of the question on any physical activity being used as a filter in the 2011 survey but not in the 2001 to 2009 surveys, we recalculate each of the above 3 variables additionally imposing the constraint that anyone who reports no physical activity on this first question is ultimately classified as inactive regardless of their responses on later questions (dashed version of all lines, appendix figure 1).

Appendix figure 1 gives the results of this comparison. By comparing the green line, which is the prevalence calculated from the BRFSS provided variable, to the orange line we see that considering total minutes of physical activity rather than number of days and duration of activity each day increases the prevalence of physical activity in the 2001 to 2009 surveys considerably. This is because a large number of people report long durations of physical activity on a small number of days. Comparing the orange line to the purple line, we see that increasing the vigorous activity threshold from 60 to 75 minutes slightly lowers the prevalence of sufficient physical activity, as is expected. Comparing the purple to the pink line, however, we see that by allowing combinations of moderate and vigorous physical inactivity, we raise the overall prevalence of sufficient physical activity, as expected. For each of the definitions, comparing the dotted line—which represents the version where an individual can only be classified as sufficiently active if they answered the question about any physical activity with 'yes'—to the non-dotted line demonstrates that this criteria lowers the prevalence of physical inactivity considerably and decreases the difference between the 2001 to 2009 surveys and the 2011 survey.

Given the obvious sensitivity of the national physical inactivity prevalence results to the definition used, we also considered how sensitive the subnational pattern of change in physical

activity is to the definition used. Appendix figure 2 depicts the change in prevalence of physical activity from 2001 to 2009 (green) and 2001 to 2011 (orange) for each state using the 6 alternative definitions given above (y-axis, one definition per panel) against the prevalence of physical activity for each state using the BRFSS calculated variables (x-axis). Change from 2001 to 2009 is robust to the definition used, likely because change calculated over this period is not affected by differences in how the physical activity data are collected in the 2011 survey compared to the 2001 to 2009 survey. With regards to change between 2001 and 2011, for most definitions, and particularly those that do not include the criteria that a respondent must report some physical activity on the initial question in order to be defined as sufficiently active, there is a shift in the magnitude of the change when comparing the alternate definitions to the BRFSS calculated variables. However, the amount of variation among the states is roughly the same, and the states that show especially poor progress (e.g. West Virginia) or especially good progress (e.g. Kentucky, Nevada) are also the same regardless of definition used.

Appendix Figure 1:
Sufficient Physical Activity Prevalence

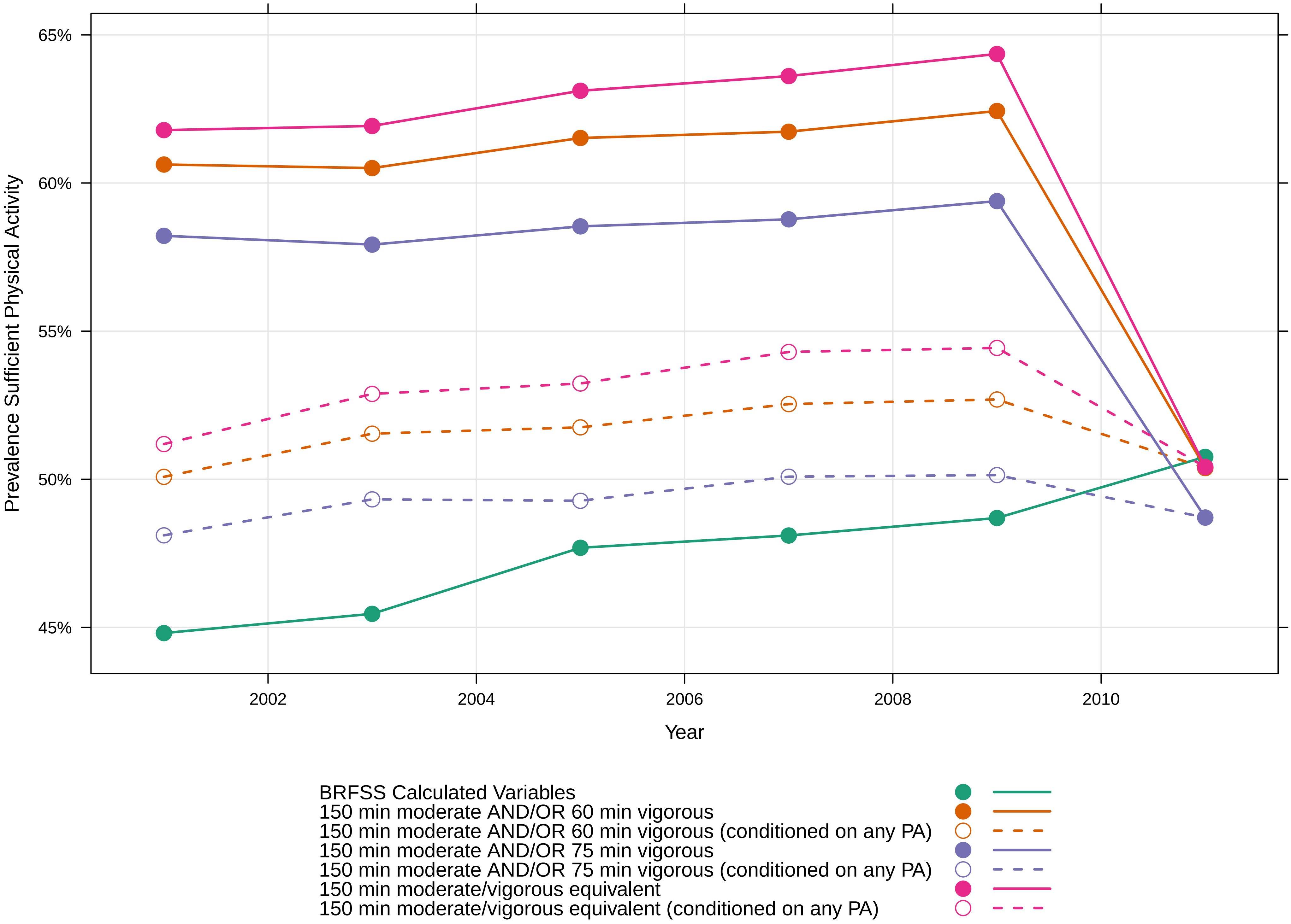

Appendix Figure 2:
Change in Sufficient Physical Activiy Prevalence by State with Different Definitions of Sufficient Physical Activity

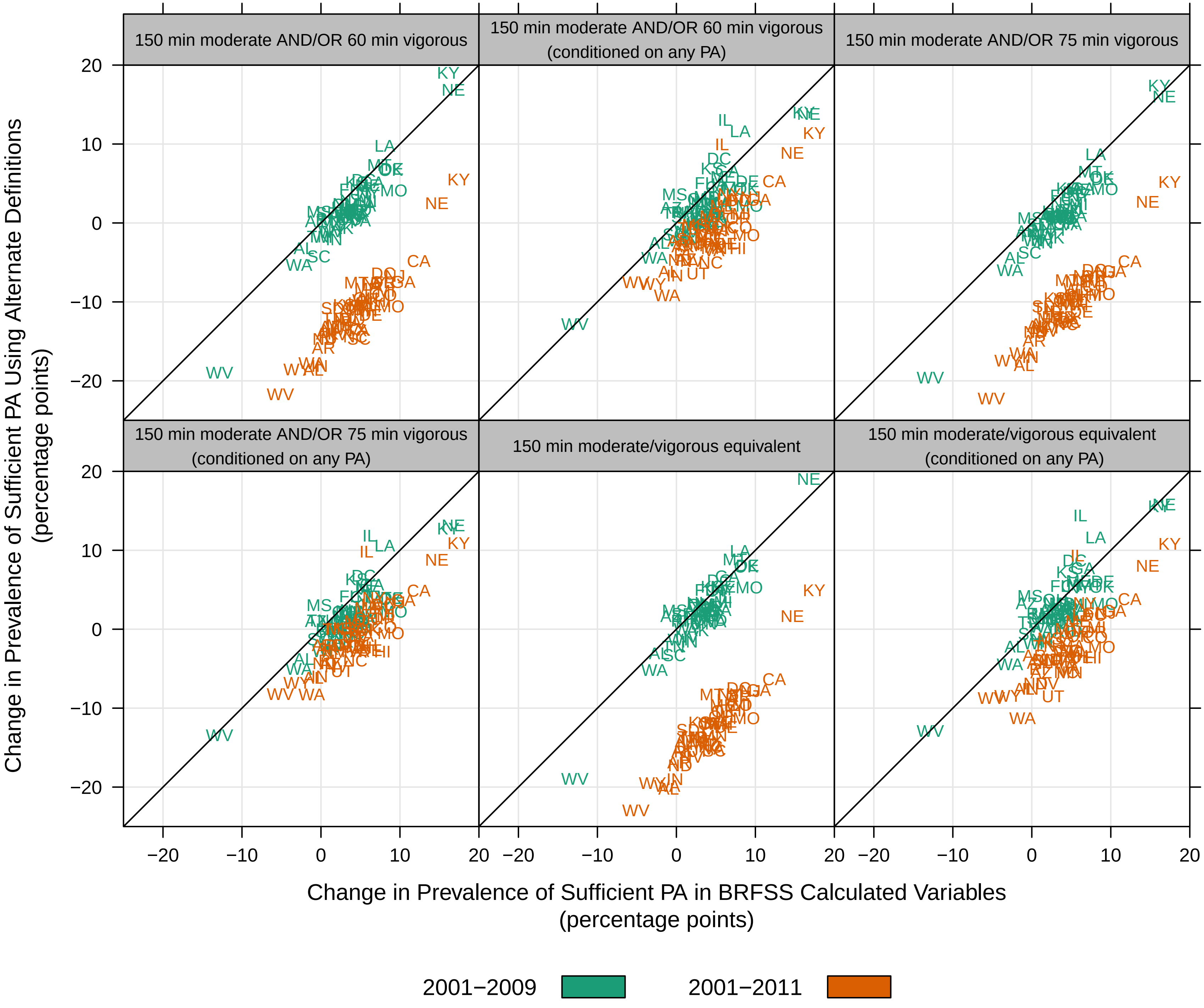

Supplement: Additional file 1 — Definition of recommended physical activity in the BRFSS and methodology with prevalence of sufficient physical activity with different definitions of physical activity and change in sufficient physical activity prevalence by state with different definitions of physical activity. [file 1478-7954-11-7-S1.pdf]
